# Outcome of haploidentical versus matched sibling donors in hematopoietic stem cell transplantation for adult patients with acute lymphoblastic leukemia: a study from the Acute Leukemia Working Party of the European Society for Blood and Marrow Transplantation

**DOI:** 10.1186/s13045-021-01065-7

**Published:** 2021-04-01

**Authors:** Arnon Nagler, Myriam Labopin, Mohamed Houhou, Mahmoud Aljurf, Ashrafsadat Mousavi, Rose-Marie Hamladji, Mohsen Al Zahrani, Sergey Bondarenko, Mutlu Arat, Emanuele Angelucci, Yener Koc, Zafer Gülbas, Simona Sica, Jean Henri Bourhis, Jonathan Canaani, Eolia Brissot, Sebastian Giebel, Mohamad Mohty

**Affiliations:** 1 Hematology Division, Chaim Sheba Medical Center, 52621 Tel Hashomer, Israel; 2 EBMT Paris Study Office, Department of Haematology, Saint Antoine Hospital, INSERM UMR 938, Sorbonne University, Paris, France; 3 ALWP of the EBMT Paris office, Paris, France; 4 King Faisal Specialist Hospital and Research Centre, Riyadh, Saudi Arabia; 5 Shariati Hospital, Hematology-Oncology and BMT Research, Tehran, Islamic Republic of Iran; 6 Service Hématologie Greffe de Moëlle, Centre Pierre Et Marie Curie, Alger, Algeria; 7 King Abdulaziz Medical City, Riyadh, Saudi Arabia; 8 Raisa Gorbacheva Memorial Institute of Children Oncology Hematology and Transplantation, First Pavlov State Medical University, Saint-Petersburg, Russian Federation; 9 Hematopoietic Stem Cell Transplantation Unit, Sisli Florence Nightingale Hospital, Istanbul, Turkey; 10 Hematology and Transplant Center, IRCCS Ospedale Policlinico San Martino, Genoa, Italy; 11 Medicana International, Istanbul, Turkey; 12 Bone Marrow Transplantation Department, Anadolu Medical Center Hospital, Kocaeli, Turkey; 13 Fondazione Policlinico A. Gemelli IRCCS, Rome, Italy; 14 Istituto di Ematologia, Università Cattolica del Sacro Cuore, Rome, France; 15 Department of Hematology, Gustave Roussy Cancer Center, Villejuif, France; 16 Hematology Division, Sheba Medical Center, Faculty of Medicine, Tel Aviv University, Tel Aviv, Israel; 17 Hematology Department, Hôpital Saint Antoine, Service d’Hématologie et Thérapie Cellulaire, Paris, France; 18 Department of Bone Marrow Transplantation and Hematology-Oncology, Maria Sklodowska-Curie National Research Institute of Oncology, Gliwice Branch, Gliwice, Poland; 19 Department of Haematology, Saint Antoine Hospital, INSERM UMR 938, Sorbonne University, Paris, France

**Keywords:** Allogeneic stem cell transplantation, Acute lymphoblastic leukemia, Haploidentical, Sibling, Donor

## Abstract

**Background:**

Non-T-cell depleted haploidentical hematopoietic stem cell transplantation (HaploSCT) is being increasingly used in acute lymphoblastic leukemia (ALL) with improving patient outcomes. We have recently reported that outcomes of adult patients (pts) with ALL in complete remission (CR) receiving HaploSCT are comparable to unrelated donor transplants. We now compared HaploSCT and matched sibling donor (MSD) transplants in pts with ALL.

**Aim:**

To assess transplantation outcomes of HaploSCT and MSD transplants in pts with ALL in CR.

**Methods:**

We retrospectively analyzed adult patients (≥ 18 years) with ALL who underwent their first allogeneic stem cell transplantation (alloSCT) in first or second CR between 2012 and 2018, either from a T cell replete Haplo or MSD donor, and whose data were reported to the Acute Leukemia Working Party (ALWP) of the European Society for Blood and Marrow Transplantation (EBMT). Multivariate analysis (MVA) adjusting for differences between the groups was performed using the Cox proportional hazards regression model. Propensity score matching was also performed to reduce confounding effects.

**Results:**

The analysis comprised 2304 patients: HaploSCT-413; MSD-1891. Median follow-up was 25 months. Median age was 37 (range 18–75) and 38 (18–76) years in HaploSCT and MSD, respectively. HaploSCT patients were transplanted more recently than those transplanted from MSD (2016 vs 2015, *p* < 0.0001). A higher rate of HaploSCT was in CR2 (33.4% vs 16.7%, *p* < 0.0001), respectively, and fewer received myeloablative conditioning (68% vs 83.2%, *p* < 0.0001). Cytomegalovirus (CMV) seropositivity was lower in HaploSCT patients (22% vs 28%, *p* = 0.01) and donors (27.1% vs 33%, *p* < 0.02), and a higher proportion of the HaploSCTs were performed using a bone marrow (BM) graft (46.2% vs 18.6%, *p* < 0.0001). The 2 groups did not differ with regard to gender, Karnofsky performance status score, ALL phenotype, Philadelphia chromosome (Ph) positivity and pre-alloSCT measurable residual disease (MRD). Graft versus host disease (GVHD) prophylaxis was mainly post-transplant cyclophosphamide (PTCy) based (92.7%) in the HaploSCT setting, while it was mostly pharmacologic in the setting of MSD (18.7% received ATG). Cumulative incidence of engraftment at day 60 was higher in MSD transplants compared to HaploSCT (98.7% vs 96.3%, *p* = 0.001), respectively. Day 180 incidence of acute (a) GVHD II-IV and III-IV was higher in HaploSCT vs. MSD: 36.3% vs 28.9% (*p* = 0.002 and 15.2% vs 10.5% (*p* = 0.005), respectively. Conversely, the 2-year chronic (c) GVHD and extensive cGVHD were 32% vs 38.8% (*p* = 0.009) and 11.9% vs 19.5% (*p* = 0.001) in HaploSCT vs MSD, respectively. Main causes of death were leukemia (31.8% vs 45%), infection (33.1% vs 19.7%) and GVHD (16.6% vs 19.7%) for HaploSCT and MSD, respectively. Two-year relapse incidence (RI), non-relapse mortality (NRM), leukemia-free survival (LFS), overall survival (OS) and GVHD-free, relapse-free survival (GRFS) were 26% vs 31.6%, 22.9% vs 13%, 51% vs 55.4%, 58.8% vs 67.4% and 40.6% vs 39% for HaploSCT and MSD, respectively. In the MVA, RI was significantly lower in HaploSCT in comparison with MSD, hazard ratio (HR) = 0.66 (95% CI 0.52–0.83, *p* = 0.004), while NRM was significantly higher, HR = 1.9 (95% CI 1.43–2.53, *p* < 0.0001). aGVHD grade II-IV and grade III-IV were higher in HaploSCT than in MSD HR = 1.53 (95% CI 1.23–1.9, *p* = 0.0002) and HR = 1.54 (95% CI 1.1–2.15, *p* = 0.011), respectively. Extensive cGVHD was lower in HaploSCT compared with MSD, HR = 0.61 (95% CI 0.43–0.88, *p* = 0.007), while total cGVHD did not differ significantly, HR = 0.94 (95% CI 0.74–1.18, *p* = 0.58). LFS, OS and GRFS did not differ significantly between the 2 transplant groups, HR = 0.96 (95% CI 0.81–1.14, *p* = 0.66); HR = 1.18 (95% CI 0.96–1.43, *p* = 0.11) and HR = 0.93 (95% CI 0.79–1.09, *p* = 0.37), respectively. These results were confirmed in a matched-pair analysis.

**Conclusions:**

Outcomes of adult patients with ALL in CR receiving alloSCT from haploidentical donors are not significantly different from those receiving transplants from MSD in terms of LFS, OS and GRFS.

**Supplementary Information:**

The online version contains supplementary material available at 10.1186/s13045-021-01065-7.

## Introduction

The common practice in treating acute leukemia is that all patients undergo human leukocyte antigen (HLA) typing at diagnosis and those with intermediate and high-risk features are referred for allogeneic stem cell transplantation (alloSCT) from an HLA matched sibling donor (MSD) if available or from an alternative donor (unrelated, haploidentical or cord blood donor) in the case where an HLA matched sibling cannot be allocated [1]. The probability of finding an HLA-identical sibling donor is estimated at 25–30% [1]. In patients lacking an HLA-identical sibling donor the next suitable option used to be matched or mismatched unrelated donors (MUD or MMUD) [1]. Subsequently, with the growing experience with HLA-mismatched family donors transplants (haploidentical stem cell transplantation) (HaploSCT) and emerging data indicating improving outcomes in this setting, HaploSCT has become a suitable alternative as indicated by the increasing numbers of HaploSCT [2]. In the last few years a multitude of clinical studies, mostly from single centers or registries, has demonstrated comparable outcomes in (HaploSCT, MUD and cord blood (CB) donors in acute leukemia, initially in acute myelogenous leukemia (AML)) [3–8] and later on in acute lymphoblastic leukemia (ALL) [9, 10]. With the increasing experience with HaploSCT, lately mainly with the non-T-depleted and post-transplantation cyclophosphamide (PTCy) approach, outcomes of HaploSCT have begun to equate to those of alloSCT from siblings which are historically considered to be the optimal stem cell graft donor [11, 12]. Recently, an analysis from the Acute Leukemia Working Party (ALWP) of the European Society for Blood and Marrow Transplantation (EBMT) demonstrated similar outcome after HaploSCT and MSD in high-risk AML, whereas in intermediate-risk AML results with sibling transplantation were superior [13]. We next compared outcomes of alloSCT from MSD to HaploSCT in patients with relapsed/refractory AML. HaploSCT was associated with inferior outcome mainly due to a higher non-relapse mortality (NRM) secondary to a high rate of infections [14]. Together with the Center for International Blood & Marrow Transplant Research (CIBMTR), we subsequently compared HaploSCT to MSD transplants in acute leukemia patients stratified by patient age. In patients aged 18 to 54 years, there were no significant differences in outcomes, while in patients aged 55 to 76 years outcome was inferior with HaploSCT from off springs in comparison with those from HLA matched siblings mostly due to higher NRM with the former donor group [15]. As for ALL, there are fewer comparisons of transplantation outcome of HaploSCT versus MSD. Data coming mainly from China indicate not only equivalent results between HaploSCT and alloSCT from MSD in patients with ALL, but moreover a reduced relapse rate, stronger graft- versus leukemia (GVL) effect, and superior attainment of measurable residual disease (MRD) negativity in HaploSCT compared with alloSCT in patients with ALL [16–18]. We therefore aimed, in the current study, to compare the outcomes of HaploSCT with those from MSD transplants in ALL, using the ALWP/EBMT registry.

## Patients and methods

### Study design and data collection

This was a retrospective, multicenter analysis using the dataset of the ALWP of the EBMT. The EBMT is a voluntary working group of more than 600 transplant centers that are required to report all consecutive stem cell transplantations and follow-ups once a year. EBMT minimum essential data forms are submitted to the registry by transplant center personnel following written informed consent from patients in accordance with center ethical research guidelines. Accuracy of data is assured by the individual transplant centers and by quality control measures such as regular internal and external audits. In addition, the study protocol was approved by each site and complied with country-specific regulatory requirements. The results of disease assessments at HCT were also submitted and form the basis of this report. Eligibility criteria for this analysis included adult patients ≥ 18 years of age with ALL in the first or second complete remission (CR1 or CR2, respectively) who underwent a first alloSCT from a haploidentical or sibling donor between 2012 and 2018. The haploidentical donor was defined as ≥ 2 HLA mismatches between donor and recipient. The exclusion criteria were alloHCT from other donor types (MUD, MMUD and umbilical CB); previous history of alloSCT; use of ex vivo T-cell-depleted hematopoietic cell graft or alemtuzumab, unknown immunophenotype or cytogenetics and advanced or unknown disease status before transplantation. Data collected included recipient and donor characteristics (age, gender, cytomegalovirus (CMV) serostatus, disease characteristics, disease status at transplant, year of transplant, and type of conditioning regimen, stem cell source, and graft versus host disease (GVHD) prophylaxis regimen). Pre-transplantation MRD status and allocation to MRD-negative or MRD-positive groups were determined by individual participating centers and utilized molecular and/or immunophenotyping criteria methodology [19]. The conditioning regimen was defined as myeloablative (MAC) or reduced intensity (RIC) based on the reports from individual transplant centers as per previously established criteria [20]. The conditioning regimen was defined as MAC when containing total body irradiation (TBI) with a dose > 6 Gy or a total dose of busulfan (Bu) > 8 mg/kg or > 6.4 mg/kg when administered orally or intravenously, respectively. All other regimens were defined as RIC. Regimens for GVHD prophylaxis were per institutional protocols. Grading of acute (a) GVHD was performed using established criteria [21]. Chronic (c) GVHD was classified as limited or extensive according to published criteria [22]. For this study, all necessary data were collected according to the EBMT guidelines, using the EBMT minimum essential data forms. The list of institutions contributing data to this study is provided in Additional file 1: Appendix.

### Statistical analysis

The study endpoints were overall survival (OS), leukemia-free survival (LFS), relapse incidence (RI), non-relapse mortality (NRM), engraftment, aGVHD, cGVHD and GVHD-free, relapse-free survival (GRFS). All endpoints were measured from the time of transplantation. Engraftment was defined as achieving an absolute neutrophil count greater than or equal to 0.5 × 10^9^/L for three consecutive days. OS was defined as time to death from any cause. LFS was defined as survival with no evidence of relapse or progression. NRM was defined as death from any cause without previous relapse or progression. We used modified GRFS criteria. GRFS events were defined as the first event among grade III–IV acute GVHD, extensive cGVHD, relapse or death from any other causes [23]. Median values and ranges were used for continuous variables and percentages for categorical variables. Patient, disease and transplant-related characteristics were compared between the two groups (HaploSCT versus MSD) using the Mann–Whitney *U* test for numerical variables, and the Chi-squared or Fisher’s exact test for categorical variables. The probabilities of OS, LFS and GRFS were calculated using the Kaplan–Meier (KM) estimate. The RI and NRM were calculated using cumulative incidence (CI) curves in a competing risk setting, death in remission being treated as a competing event for relapse. Early death was considered as a competing event for engraftment. To estimate the CI of acute or chronic GVHD, relapse and death were considered as competing events. Univariate analyses were performed using the log-rank test for LFS and OS, while Gray’s test was used for CI. Multivariate analyses were performed using the Cox proportional hazards regression model. All variables differing significantly between the two groups and potential risk factors were included in the model. Results were expressed as the hazard ratio (HR) with a 95% confidence interval (95% CI). Finally, a propensity score (PS) matched pairs analysis was conducted to corroborate the results obtained in the global population. Each patient identified as having received HaploSCT was matched with a patient who had received one from a MSD. PS was based on patient age and sex, ALL phenotype, Philadelphia chromosome (Ph)-negative B ALL/Ph-positive B ALL/T-ALL), status at transplantation, conditioning (MAC-TBI, MAC-chemotherapy and RIC), cell source (bone marrow (BM), peripheral blood (PB)) and patient and donor CMV. Matched control on PS was defined as exact matching for diagnosis and status at transplantation and nearest neighbor for other variables. Patient was well matched with standardized mean difference estimates of less than 5% for all parameters. In order to test for a center effect, we introduced a random effect or frailty for each center into the model [24]. All *p* values were two-sided with a type 1 error rate fixed at 0.05. Statistical analyses were performed with SPSS 24.0 (SPSS Inc., Chicago, IL, USA) and R 3.4.1. Analyses were performed using the R statistical software version 3.2.3 (available online at http://www.R-project.org) and propensity score analysis using the package ‘MatchIt.’

## Results

### Patient, transplant and disease characteristics

A total of 2304 patients met the inclusion criteria, 413 in the HaploSCT and 1891 in the MSD cohorts. The median duration of follow-up from alloSCT was 25 months for the entire study population. HaploSCT was performed more recently with a median year of transplant of 2016 compared with MSD, median year of transplant 2015 (Table 1). The primary diagnosis was Ph-negative B-ALL, Ph-positive B-ALL and T-ALL in 148 (35.8%), 151 (36.6%), and 114 (27.6%) and 616 (32.6%), 725 (38.3%) and 550 (29.1%) patients undergoing HaploSCT and MSD transplants, respectively. As for disease status, significantly more patients were in CR1 rather than in CR2 before transplant with either Haplo or MSD transplants. The disease status before alloSCT was CR1 and CR2 in 275 (66.6%) and 138 (33.4%) and 1575 (83.3%) and 316 (16.7%) of HaploSCT and MSD patients, respectively (*p* < 0.0001).

**Table 1:** Baseline patient, donor and disease characteristics at diagnosis

Clinical parameter	MSD (*n* = 1891)	Haplo (*n* = 413)	*p*
Follow-up duration in m, median (range)	24.88 (12.13;46.05)	25.37 (13.21;42.56)	0.92
Age at transplant in years, median (range)	37.7 (18–76.1) [27–49.5]	37.1 (18.1–75) [25.7–51]	0.58
Year of transplant	2015 (2012–2018)	2016 (2012–2018)	< 0.0001
*Patient gender, n (%)*			
Male	1129 (59.8%)	268 (64.9%)	0.055
Female	759 (40.2%)	145 (35.1%)	
*Diagnosis*			
Ph-neg B ALL	616 (32.6%)	148 (35.8%)	0.44
Ph-pos B ALL	725 (38.3%)	151 (36.6%)	
T-ALL	550 (29.1%)	114 (27.6%)	
*Patient CMV status*			
Patient CMV negative, n (%)	521 (28.1%)	90 (22%)	0.01
Patient CMV positive, n (%)	1335 (71.9%)	319 (78%)	
*Donor CMV status*			
Donor CMV negative, n (%)	599 (33%)	110 (27.1%)	0.021
Donor CMV positive, n (%)	1215 (67%)	296 (72.9%)	
*Status at transplant*			
CR1	1575 (83.3%)	275 (66.6%)	< 0.0001
CR2	316 (16.7%)	138 (33.4%)	
*KPS at transplant, n (%)*			
< 90	397 (22.4%)	100 (25%)	0.27
≥ 90	1373 (77.6%)	300 (75%)	
Missing	121	13	
Donor age, y median (range)	38.5 (0.1–74) [27.5–50.5]	38.8 (7.8–74.3) [27.1–49.3]	0.79
*Donor gender*			
Male donor	1033 (54.9%)	242 (58.6%)	0.17
Female donor	848 (45.1%)	171 (41.4%)	
*Donor-recipient gender matching, n (%)*			
Female–male	474 (25.2%)	110 (26.6%)	0.54
Other	1408 (74.8%)	303 (73.4%)	
*MRD at transplant*			
Negative	681 (67.4%)	162 (65.3%)	0.54
Positive	330 (32.6%)	86 (34.7%)	
Missing	880	165	

KPS, Karnofsky performance status;TBI, total body irradiation; CMV, cytomegalovirus; MRD, measurable residual disease

Pre-alloSCT MRD was comparable between the 2 groups with 65.3% and 67.4% MRD negativity for HaploSCT and MSD transplants, respectively (Table 1). The use of RIC was more frequent in the HaploSCT patients, 132 (32%) compared with the MSD patients 317 (16.8%), respectively, while MAC was more frequently used in patients undergoing MSD transplants: 83.2% vs 68%, respectively (*p* < 0.0001). Similarly, TBI was more frequently used in patients undergoing MSD: 64.9% vs 42.6% (*p* < 0.0001), respectively (Table 2). As for MSD the most common MAC regimen was Bu/Flu and Bu/Cy (19.3%), while the most common RIC regimen was Flu/TBI with or without Cy (9.6%). A full list of conditioning regimens is provided in Additional file 2: Table S1. Bone marrow (BM) was more frequently used graft source in the HaploSCT cohort compared with the MSD cohort: 46.2%vs 18.6%, while mobilized PB cells (PBSC) were used more often in MSD transplants: with 81.4% vs (53.8% for MSD vs HaploSCT, respectively (*p* < 0.0001)). PTCy was the most common anti-GVHD prophylaxis used in the HaploSCT setting 383 (92.7%), while cyclosporine A plus methotrexate (no PTCy regimen) was the most common GVHD prophylaxis regimen for MSD 1273 (67.3%). Anti-thymocyte globulin (ATG) was used in 61 (14.8%) and 354 (18.7%) of the HaploSCT and MSD cohorts, respectively (*p* = 0.058), Table 2. The rest of the patient and transplant-related characteristics were comparable between groups.

**Table 2:** Transplant characteristics

Clinical parameter	MSD (*n* = 1891)	Haplo (*n* = 413)	*p*
*Conditioning intensity*			
Myeloablative conditioning, n (%)	1574 (83.2%)	281 (68%)	< 0.0001
Reduced intensity conditioning, n (%)	317 (16.8%)	132 (32%)	
*TBI*			
CT	663 (35.1%)	237 (57.4%)	< 0.0001
TBI	1228 (64.9%)	176 (42.6%)	
*Graft source*			
Bone marrow	351 (18.6%)	191 (46.2%)	< 0.0001
Peripheral blood	1540 (81.4%)	222 (53.8%)	
*PTCy*			
No PTCy	1812 (95.8%)	30 (7.3%)	< 0.0001
PTCy	79 (4.2%)	383 (92.7%)	
*In vivo T-cell depletion*			
No ATG	1537 (81.3%)	352 (85.2%)	0.058
ATG	354 (18.7%)	61 (14.8%)	

MSD: matched sibling donor, Haplo: haploidentical transplantation, MAC: myeloablative conditioning, RIC: reduced intensity conditioning, TBI: total body irradiation; BM: bone marrow; PB: mobilized peripheral blood stem cells; PTCy: post-transplantation cyclophosphamide; ATG: anti-thymocyte globulin; MAC: myeloablative conditioning; TBI: total body irradiation; CT: chemotherapy; RIC: reduced intensity conditioning

### Transplantation outcomes

Table 3 shows the cumulative incidence of engraftment at day 60, with a higher rate noted in MSD recipients compared to the HaploSCT group: 98.9% versus 96.5%, *p* < 0.0001, respectively, with a shorter median time to engraftment in this group (16 versus 18 days in HaploSCT, *p* < 0.01). Similarly, non-engraftment was higher in the HaploSCT 3.5% vs 1.1% in the MSD transplants, respectively, *p* < 0.0001. Of note, engraftment rate was higher following PBSC compared to BM in the HaploSCT group 94.6% vs 97.7%, respectively (*p* < 0.037). Day 180 incidence of grade II-IV and III-IV aGvHD was higher in HaploSCT compared to MSD 35.6% vs 28.1%, *p* = 0.002 and 15.2% vs 10.5%, *p* = 0.009, respectively.

**Table 3:** Transplant outcomes at day 180

Clinical parameter	MSD (*n* = 1891)	Haplo (*n* = 413)	*p*
*Engraftment*			
Graft failure	21 (1.1%)	14 (3.5%)	< 0.0001
Engrafted	1831 (98.9%)	387 (96.5%)	
Missing	39	12	
*Acute GVHD*			
Grade I	266 (14.5%)	67 (17%)	0.009
Grade II	329 (17.9%)	81 (20.6%)	
Grade III	132 (7.2%)	42 (10.7%)	
Grade IV	55 (3%)	17 (4.3%)	
Present, grade unknown	34 (1.8%)	6 (1.5%)	
No aGvHD present (grade 0)	1023 (55.6%)	181 (45.9%)	
Missing	52	19	

HSCT hematopoietic stem cell transplantation; GVHD graft versus host disease

As indicated in Table 4, the 2-year cGvHD and extensive cGVHD rates were lower in HaploSCT as compared to MSD 32% vs 38.8%, *p* = 0.009 and 11.9% vs 19.5%, *p* = 0.001. At 2 years, RI was 26% vs 31.6% (*p* = 0.017) and NRM was 22.9% vs 13% (*p* < 0.001) in HaploSCT and MSD recipients, respectively. The probability of LFS and OS was 55.4% *versus* 51% (*p* = 0.07) and 58.8% *versus* 67.4% (*p* < 0.001) in HaploSCT and MSD, respectively. The incidence of GRFS was 40.6% *versus* 39% (*p* = 0.74), respectively.

**Table 4:** Univariate analysis of 2-year clinical outcomes

	RI	NRM	LFS	OS	GRFS	aGVHD grade II–IV	aGVHD grade III–IV	cGVHD	ext. cGVHD
MSD	31.6% [29.2–34%]	13% [11.4–14.7%]	55.4% [52.8–57.9%]	67.4% [64.8–69.8%]	39% [36.4–41.5%]	28.9% [26.8–31.1%]	10.5% [9.1–12%]	38.8% [36.3–41.3%]	19.5% [17.5–21.7%]
Haplo	26% [21.5–30.8%]	22.9% [18.8–27.3%]	51% [45.7–56.2%]	58.8% [53.3–63.9%]	40.6% [35.4–45.7%]	36.3% [31.5–41.1%]	15.2% [11.9–19%]	32% [27.1–37.1%]	11.9% [8.6–15.8%]
*p*	0.017	0.001	0.07	0.001	0.74	0.002	0.005	0.009	0.001

RI, relapse incidence; NRM, non-relapse mortality; LFS, leukemia-free survival; OS, overall survival; GVHD, graft versus host disease; GRFS, GVHD-free/relapse-free survival; KPS, Karnofsky performance status; ATG, anti-thymocyte globulin; PTCy, post-transplant cyclophosphamide; RIC, reduced intensity conditioning; MAC, myeloablative conditioning; PB, peripheral blood; BM, bone marrow

### Cause of death

Additional file 2: Table S2 shows the cause of death. A total of 161 (39%) patients in the HaploSCT cohort and 573 (30%) patients in the MSD cohort died during the study period. Disease relapse was the most common cause of death in both HaploSCT (31.8%) and MSD (45%) cohorts. Infection-related deaths were more common in the HaploSCT cohort (33.1% vs. 19.7%). Rates of GVHD-related deaths were similar between the HaploSCT and MSD cohorts (16.6% vs. 19.7%), respectively. Deaths due to veno-occlusive disease (VOD) of the liver and multiorgan failure (MOF) were also similar in magnitude between the HaploSCT and MSD cohorts: 3.2% versus 3.5% and 3.8% versus 5%, respectively, while interstitial pneumonitis (IP) accounted for 3.2% and 2.4% of the death, respectively.

### Multivariate analysis

In MVA (Table 5) RI was significantly lower in HaploSCT in comparison with MSD (*p* = 0.004), while NRM was significantly higher (*p* < 0.0001). aGVHD grade II-IV and grade III-IV were higher in HaploSCT than in MSD (*p* = 0.0002 and *p* = 0.011), respectively. The incidence of extensive cGVHD was lower in HaploSCT compared with MSD (*p* = 0.007), while total cGVHD did not differ significantly (*p* = 0.58). Rates of LFS, OS and GRFS did not differ significantly between HaploSCT and MSD transplants.

**Table 5:** Multivariate analysis

	RI		NRM		LFS		OS		GRFS	
	HR (95% CI)	*P*	HR (95% CI)	*P*	HR (95% CI)	*P*	HR (95% CI)	*P*	HR (95% CI)	*P*
Haplo vs MSD	0.66 (0.52–0.83)	0.0004	1.9 (1.43–2.53)	< 0.0001	0.96 (0.81–1.14)	0.66	1.18 (0.96–1.43)	0.11	0.93 (0.79–1.09)	0.37
Age (per 10 years)	1.01 (0.94–1.08)	0.87	1.33 (1.21–1.47)	< 0.0001	1.1 (1.05–1.17)	0.0004	1.19 (1.12–1.27)	< 0.0001	1.11 (1.06–1.08)	< 0.0001
*Ph-neg B-ALL (ref)*										
Ph-pos B-ALL	0.77 (0.63–0.95)	0.012	1.15 (0.86–1.54)	0.34	0.88 (0.75–1.14)	0.12	0.74 (0.61–0.9)	0.002	0.94 (0.82–1.08)	0.4
T-ALL	1.04 (0.85–1.26)	0.73	1.19 (0.87–1.62)	0.28	1.07 (0.91–1.26)	0.43	1.13 (0.94–1.36)	0.19	1 (0.86–1.15)	0.95
CR2 vs CR1	2.5 (2.08–3)	< 0.0001	1.98 (1.5–2.62)	< 0.0001	2.31 (1.99–2.7)	< 0.0001	2.57 (2.17–3.06)	< 0.0001	1.95 (1.69–2.24)	< 0.001
Year of HSCT	0.99 (0.95–1.03)	0.64	0.94 (0.89–1.01)	0.074	0.98 (0.94–1.01)	0.17	0.96 (0.92–1)	0.082	1 (0.97–1.03)	0.98
Female to male vs. other	0.96 (0.8–1.15)	0.65	1.22 (0.94–1.57)	0.14	1.03 (0.88–1.19)	0.73	1.13 (0.95–1.34)	0.16	1.27 (1.12–1.45)	0.0002
MAC TBI (reference)										
MAC CT	1.32 (1.08–1.61)	0.006	1.03 (0.76–1.39)	0.87	1.22 (1.04–1.44)	0.016	1.1 (0.9–1.34)	0.34	1.05 (0.9–1.23)	0.54
RIC	1.61 (1.27–2.02)	< 0.0001	0.62 (0.43–0.88)	0.007	1.18 (0.97–1.43)	0.095	0.99 (0.79–1.24)	0.92	1.02 (0.86–1.22)	0.82
Patient CMV positive	0.96 (0.79–1.18)	0.72	1.59 (1.15–2.19)	0.005	1.12 (0.95–1.33)	0.18	1.31 (1.07–1.6)	0.009	1.13 (0.97–1.31)	0.11
Donor CMV positive	1.21 (1.99–1.49)	0.063	0.86 (0.65–1.15)	0.32	1.09 (0.92–1.29)	0.3	1.01 (0.84–1.23)	0.89	1.01 (0.87–1.16)	0.94
Center (frailty)		0.32		0.031		0.31		0.064		0.007

	Acute GVHD grade II–IV		Acute GVHD grade III–IV		Chronic GVHD		Extensive chronic GVHD	
	HR (95% CI)	*P*	HR (95% CI)	*P*	HR (95% CI)	*P*	HR (95% CI)	*P*
Haplo vs MSD	1.53 (1.23–1.9)	0.0002	1.54 (1.1–2.15)	0.011	0.94 (0.74–1.18)	0.58	0.61 (0.43–0.88)	0.007
Age (per 10 years)	1.03 (0.96–1.1)	0.43	1.08 (0.97–1.2)	0.15	1.11 (1.03–1.18)	0.005	0.17 (1.06–1.29)	0.002
*Ph-neg B-ALL (ref)*								
Ph-pos B-ALL	0.93 (0.76–1.114)	0.49	1.15 (0.83–1.58)	0.4	0.82 (0.67–0.99)	0.041	1.03 (0.79–1.35)	0.83
T-ALL	0.88 (0.71–1.09)	0.24	0.94 (0.66–1.33)	0.73	0.84 (0.69–1.04)	0.11	0.99 (0.74–1.32)	0.94
CR2 vs CR1	1.11 (0.9–1.37)	0.31	1.34 (0.97–1.84)	0.078	1.25 (1–1.57)	0.051	1.55 (1.13–2.13)	0.006
Year of HSCT	0.96 (0.92–1)	0.053	1 (0.93–1.07)	0.96	0.96 (0.92–1)	0.044	1.04 (0.98–1.1)	0.23
Female to male vs. other	1.23 (1.02–1.47)	0.028	1.36 (1.03–1.82)	0.033	1.73 (1.46–2.05)	< 0.0001	1.98 (1.57–2.5)	< 0.0001
MAC TBI (reference)								
MAC CT	0.82 (0.65–1.04)	0.1	1.25 (0.9–1.75)	0.19	0.78 (0.62–0.98)	0.035	0.69 (0.49–0.96)	0.03
RIC	0.78 (0.61–1.01)	0.056	0.79 (0.53–1.19)	0.26	0.81 (0.63–1.04)	0.1	0.72 (0.5–1.04)	0.078
Patient CMV positive	1.01 (0.82–1.25)	0.93	1.34 (0.94–1.91)	0.1	1.04 (0.85–1.27)	0.69	1.17 (0.89–1.54)	0.27
Donor CMV positive	1.04 (0.85–1.28)	0.69	0.78 (0.56–1.08)	0.13	1.05 (0.86–1.27)	0.63	1.01 (0.77–1.31)	0.97
Center (frailty)		< 0.0001		0.1		< 0.0001		< 0.0001

RI, relapse incidence; NRM, non-relapse mortality; LFS, leukemia-free survival; OS, overall survival; GVHD, graft versus host disease; GRFS, GVHD-free/relapse-free survival; MRD, minimal residual disease; ATG, anti-thymocyte globulin; PTCy, post-transplant cyclophosphamide; RIC, reduced intensity conditioning; MAC, myeloablative conditioning; PB, peripheral blood; BM, bone marrow

Other significant prognostic factors in the MVA for higher risk of RI were disease status of CR2, RIC and non-TBI (MAC), while Ph + ALL was a significant prognostic factor for a lower RI. Significant prognostic factors for higher NRM rate were increasing age, disease status of CR2 and patient CMV positivity. RIC predicted lower NRM.

Rates of grade II-IV aGVHD and severe aGVHD increased with increasing age and female donors to male patients, while for total and extensive cGVHD it was chemotherapy-based MAC. Increasing age and disease status (CR2 vs CR1) were additional prognostic factors predictive of a higher risk of extensive cGVHD.

### Propensity score matching analysis

**Fig. 1:**
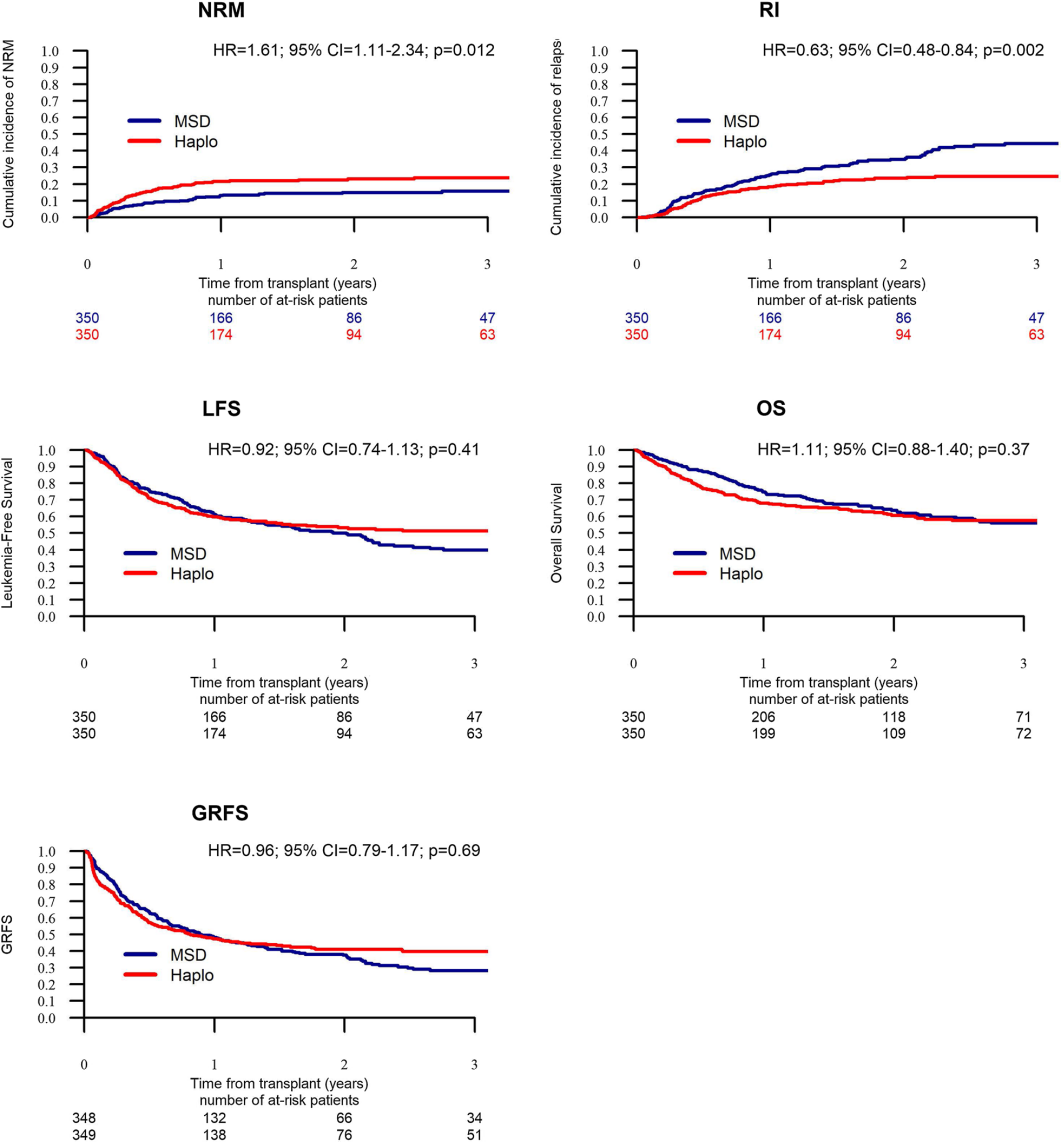
Matched-pair analysis of transplantation outcome—non-relapse mortality (NRM), relapse incidence (RI), leukemia-free survival (LFS), overall survival (OS) and GVHD-free, relapse-free survival (GRFS) in allogeneic stem cell transplantation from haploidentical (Haplo) donors and matched sibling donors (MSD)

We were able to pair-match 350 HaploSCT with 350 MSD (Additional file 2: Tables S3–S5). The results were consistent with the results of the MVA. HaploSCT was associated with a higher risk of NRM (*p* < 0.012), lower RI (*p* < 0.002) and similar rates of LFS (*p* = 0.41), OS (*p* = 0.41) and GRFS (*p* = 0.69) (Additional file 2: Table S6). Survival curves according to the results of the matched pair analysis are shown in Fig. 1. Acute GVHD incidence was higher in HaploSCT in comparison with MSD transplants, while incidence of severe aGVHD was not significantly different. Chronic GVHD incidence was similar between HaploSCT and MSD, while extensive cGVHD was lower with HaploSCT vs MSD transplants. Causes of death were similar between the two groups (Additional file 2: Table S7).

## Discussion

Allogeneic stem cell transplantation is a curative option in patients with ALL and is the treatment of choice in patients in CR1 with high-risk features and in patients with CR2 [25, 26, 28, 29]. Historically, sibling donors constituted the traditional donor pool and were considered to be the optimal and preferred donor if available [1]. In recent years, the number of haploidentical transplants is increasing and results are improving including in ALL [2, 8, 27], and thus in the absence of an MSD, HaploSCT may represent a valid alternative [9, 10, 28]. In the current study, we retrospectively analyzed and compared survival and other transplant-related outcomes of patients with ALL who underwent allogeneic transplantation from haploidentical versus sibling donors.

Our results indicate that compared with MSD recipients, HaploSCT recipients had a somewhat lower incidence of neutrophil recovery and a higher incidence of graft failure, in line with some of the previous studies in patients with AML [3, 13, 14] and recently in ALL [16]. This is probably due to the higher proportion of patients receiving BM grafts among HaploSCT [3]. Indeed**,** engraftment was better following PBSC compared to BM in the HaploSCT group. In agreement with some of the previous publications the incidence of aGVHD and severe aGVHD was higher following HaploSCT compared with MSD transplants [13, 14], while other publications comparing HaploSCT to MSD transplants in ALL have reported a similar incidence of aGVHD [16, 17]. As previously reported, female donors to male patients and center effect were associated with a higher rate of aGVHD [29]. The high incidence of aGVHD following HaploSCT in combination with slow immune recovery most probably led to a higher incidence of infection-related deaths and overall higher transplant-related mortality observed in HaploSCT compared to MSD in agreement with previous publications [14, 15, 29]. The incidence of cGVHD did not differ significantly between HaploSCT and MSD, while the frequency of extensive cGVHD was lower as reported in some of the previous studies comparing HaploSCT to MSD transplants in AL and AML and recently in ALL and may be related to the higher proportion of BM grafts in the haploidentical setting [3, 18, 30]. In accordance with the foregoing, we recently compared BM to PB grafts for HaploSCT in ALL demonstrating a lower incidence of GVHD with BM grafts [31]. As for the discrepant results with acute and chronic GVHD it may speak to the different pathophysiology between the two but moreover may be due to the different anti-GVHD prophylaxis used in the HaploSCT vs MSD transplant setting and especially the PTCy used almost exclusively in the HaploSCT group. Additional prognostic factors for cGVHD were female donor to a male patients and center effect as well as age, year of transplant, ALL phenotype and chemotherapy-based MAC. The increased incidence of GVHD with both increasing age and MAC and the improvement of GVHD outcome in more recent alloSCTs have been reported previously [32, 33]. As for RI it was significantly lower in HaploSCT vs MSD transplants. It is conceivable at least from a theoretical standpoint that due to the broader HLA disparity in HaploSCT, the GVL effect is stronger in haploidentical compared with that of allogeneic transplantation from a sibling. We have recently observed a lower incidence of AML relapse with HaploSCT compared to MSD transplants with PTCy as GVHD prophylaxis [36], while we failed to demonstrate stronger GVL in haploidentical versus sibling allogeneic transplantation including in second allogeneic transplantation with haploidentical donors or broader GVHD prophylaxis protocols [33, 34]. The magnitude of the GVL post-HaploSCT and thus post-transplantation RI may have to do with the type of anti-GVHD prophylaxis used [35]. As for ALL, Chen et al. explored the incidence, risk factors and outcomes of central nervous system (CNS) relapse as well as systemic relapses post-alloSCT in 1970 ALL patients from haploidentical (*n* = 1586) and MSD transplants (*n* = 336), respectively [16]. The cumulative incidence of CNS relapse did not differ -3.91% and 5.36% in HaploSCT and MSD transplants, respectively. Similarly, the 3-year cumulative incidence of systemic relapse was also comparable between the two subgroups (HaploSCT 40.6 ± 7.4%; MSD 13.3 ± 8.7%, respectively, *p* = 0.085). In contrast Chang et al. recently reported on a prospective genetically randomized study in ALL comparing transplantation outcome of HaploSCT (*n* = 169) and MSD (*n* = 39). The three-year RI was significantly lower post-HaploSCT in comparison with MSD (HR  0.364; *p* = 0.001) [17]. Similar findings were reported by Li et al. [18] comparing HaploSCT (*n* = 166) to MSD (*n* = 36) in Ph + ALL demonstrating a lower RI with HaploSCT of 14.8% vs 56.4% [17]. Importantly, in a very elegant recent paper Prof Xiao Jun Huang's group studied the immune cell dynamic response during leukemia development in a mouse model elucidating the immunological mechanism behind the stronger GVL in HaploSCT versus MSD transplants demonstrating decreased apoptosis and increased cytotoxic cytokine secretion by T and natural killer (NK) cells in the Haplo transplantation model [36]. Moreover, from a theoretical standpoint the strong anti-leukemia activity of the haploidentical allograft could be translated into superior survival by decreasing the NRM [37]. Of note, in our previous studies comparing HaploSCT to transplantation from MUD or MMUD in ALL we observed similar transplantation outcome including NRM and RI [9, 10]. The difference as for NRM and RI in the HaploSCT vs MUD transplants comparison compared to the current HaploSCT vs MSD transplants comparison may be due to the broader HLA disparity in MUD vs MSD translated into stronger GVL effect but also higher TRM [38].

Overall, our analysis confirmed by PS (in order to balance characteristics of the two populations) shows that outcomes of alloSCT from haploidentical donors were comparable to MSD transplants for ALL patients demonstrating similar LFS, OS and GRFS. These results are in agreement with two recent publications comparing HaploSCT vs MSD transplants in patients with ALL from China with the Chinese pioneered haploidentical protocol and in a younger age group compared with our cohort [17, 39]. The fact that the outcome of haploidentical transplantation is similar to that of sibling transplantation, which is still held to be the donor gold standard, underscores the major improvement of the HaploSCT over the years [8] and is of major clinical importance as it will ensure the speed and chance of finding a donor and moreover will give the transplanting physician the option to choose the optimal donor for a specific patient [40], an opportunity that is usually not available in allogeneic transplantation from sibling donors. The use of HaploSCT in a hematological malignancy like ALL will afford the distinct advantage of a readily available pool of related donors avoiding the potentially hazardous delay caused by the search for a HLA matched donor and further capitalizing on the relative abundance of possible donors available for prospective HaploSCT patients. This will potentially allow for refinement of donor selection with the aim of matching patients with the optimal compatible donor. Additional prognostic factors for LFS, OS and GRFS in our analysis were increasing age, disease status (CR2) as well as chemotherapy-based conditioning for LFS, while Ph + ALL and patient CMV status were additional prognostic factors for OS. As for GRFS, additional significant factors were female donor to a male patient and center effect. Female donor to a male patient and center effect were also significant for GVHD. These factors are known and concur with previously reported factors for transplantation outcome in AL in general, including ALL.

This being a retrospective and registry-based study, there are several limitations including the possibility of unavailable data that have not been considered, missing MRD data as well as prior pre-HSCT lines of therapy including tyrosine kinase inhibitors for Ph + ALL. However, the MRD data were relatively equally distributed among the two groups. In addition, as for performance status and transplantation risk we had the KPS scores but lacked the transplantation comorbidity index. Lastly, we note that our study did not use the NIH consensus criteria for cGVHD grading.

## Conclusions

In aggregate, in this large registry-based retrospective analysis outcomes of patients with ALL undergoing transplantation from a haploidentical donor were comparable with those undergoing MSD with similar LFS, OS and GRFS. Prospective intention-to-treat studies are required to confirm these results.

## Supplementary Information

**Additional file 1:** Appendix.

**Additional file 2:** Supplementary tables.

## Data Availability

Not applicable.
