# Supplementary material for: Outcome of haploidentical versus matched sibling donors in hematopoietic stem cell transplantation for adult patients with acute lymphoblastic leukemia: a study from the Acute Leukemia Working Party of the European Society for Blood and Marrow Transplantation
Source: J Hematol Oncol. 2021 Apr 1;14:53. doi: 10.1186/s13045-021-01065-7 (PMC8017786; doi:10.1186/s13045-021-01065-7)
Supplement: Supplementary file 1 — Additional file 1: Appendix. [file 13045_2021_1065_MOESM1_ESM.docx]

Appendix Supplementary material: Contributing Centers

King Faisal Specialist Hospital & Research Centre, Oncology (Section of Adult Haematolgy/BMT), Riyadh, Saudi Arabia; Shariati Hospital, Hematology-Oncology and BMT Research, Teheran, Iran; Centre Pierre et Marie Curie, Service Hématologie Greffe de Moëlle, Alger, Algeria; King Abdul - Aziz Medical City, Riyadh, Saudi Arabia; Demiroglu Bilim University Istanbul Florence Nightingale Hospital, Hematopoietic SCT Unit, Istanbul, Turkey; Programme de Transplantation&Therapie Cellulaire, Centre de Recherche en Cancérologie de Marseille, Institut Paoli Calmettes, Marseille, France; Hopital St. Louis, Dept.of Hematology - BMT, Paris, France; Techniciens d`Etude Clinique suivi de patients greffes, Nouvel Hopital Civil, Strasbourg, France; First State Pavlov Medical University of St. Petersburg, Raisa Gorbacheva Memorial Research Institute for Paediatric Oncology, Hematology, and Transplantation, St Petersburg, Russia; First Affiliated Hospital of Soochow University, Department of Hematology, Suzhou, China; Gustave Roussy Cancer Campus, BMT Service, Department of Hematology,, Villejuif, France; King Hussein Cancer Centre, Queen Rania Street - Aljubiha, Amman, Jordan; Dél-pesti Centrumkórház –, Országos Hematológiai és Infektológiai Intézet, Dept. Haematology and Stem Cell Transplant, Budapest, Hungary; CHU de Lille, LIRIC, INSERM U995, Université de Lille, Lille, France; Centre Hospitalier Lyon Sud, Pavillon Marcel Bérard -Bat 1G, Service Hematologie, Lyon, France; University Hospital, Hematology, Basel, Switzerland; Institute of Hematology and Blood Transfusion, Servicio de Hematología, Prague, Czech Republic; Ospedale San Martino, Department of Haematology II, Genova, Italy; University Hospital Center Rebro, Kispaticeva 12, Zagreb, Croatia; Department of Internal Medicine, American University of Beirut Medical Center, Beirut, Lebanon; HUCH Comprehensive Cancer Center, Stem Cell Transplantation Unit, Helsinki, Finland; University Hospital La Fe, Hematology Department (Torre F, Planta 7), Valencia, Spain; Medicana International Hospital Istanbul, Adult BMT Unit, Istanbul, Turkey; University Hospital, Dept. of Bone Marrow Transplantation, Essen, Germany; Ospedale San Gerardo, Clinica Ematologica dell`Universita Milano-Biocca, Monza, Italy; Hopital Saint Antoine, Department of Hematology, Paris, France; Erasmus MC Cancer Institute, University Medical Center Rotterdam, Department of Hematology, Rotterdam, The Netherlands; CHU Nantes, Dept. D`Hematologie, Nantes, France; Baskent University Hospital, Haematology Division, BMT Unit, Haemaology Reserach Laboratory, Training & Medical, Adana, Turkey; George Papanicolaou General Hospital, Haematology Department / BMT Unit, Thessaloniki, Greece; Imperial College, Department of Haematology, Hammersmith Hospital, London, United Kingdom; Universite Paris IV, Hopital la Pitié-Salpêtrière, Hematologie Clinique, Paris, France; CHU Bordeaux, Hôpital Haut-leveque, Pessac, France; CHU Grenoble Alpes - Université Grenoble Alpes, Service d`Hématologie, Grenoble, France; CHU Lapeyronie, Département d`Hématologie Clinique, Montpellier, France; Hosp. Reina Sofia, Córdoba Hospital, Department of Hematology, Cordoba, Spain; Goethe-Universitaet, Medizinische Klinik II, Hämatologie, Medizinische Onkologie, Frankfurt Main, Germany; Klinikum Grosshadern, Med. Klinik III, Munich, Germany; Gazi University Faculty of Medicine, Hematology, Ankara, Turkey; Karolinska University Hospital, Dept. of Hematology, Stockholm, Sweden; Ospedale Civile, Dipartimento Oncologico Ematologico, Pescara, Italy; Universita Cattolica S. Cuore, Istituto di Ematologia, Ematologia, Rome, Italy; University of Heidelberg, Medizinische Klinik u. Poliklinik V, Heidelberg, Germany; Centre Hospitalier Universitaire de Rennes, Service d`Hematologie Clinique Adulte, Rennes, France; Hadassah University Hospital, Dept. of Bone Marrow Transplantation, Jerusalem, Israel; CHRU BRABOIS, Service Hématologie, Vandoeuvre Nanc, France; Azienda Ospedaliero Universitaria di Udine, Division of Hematology, Udine, Italy; Hospital Universitario Virgen del Rocío, Servicio de Hematologia y Hemoterapia, Servicio Andaluz de Salud, Sevilla, Spain; USD Trapianti di Midollo, Adulti, Universita di Brescia, Brescia, Italy; University Hospital Gasthuisberg, Dept. of Hematology, Leuven, Belgium; Hospital Sirio-Libanes, Hematology Bone Marrow Transplant Unit, Sao Paulo, Brazil; BMT unit, Clinica Ematologica, Fondazione IRCCS Policlinico San Matteo, Pavia, Italy; ASST GRANDE OSPEDALE METROPOLITANO NIGUARDA, Hematology Department, Milano, Italy; U.O.D Trapianti di midollo osseo, A.O.R Villa Sofia-Cervello, Palermo, Italy; ASST Papa Giovanni XXIII, Hematology and Bone Marrow Transplant Unit, Bergamo, Italy; Centro Trapianti Unico Di CSE Adulti e Pediatrico A. O Brotzu, Via Edward Jenner, 1, Cagliari, Italy; Hospital Gregorio Marañón, Sección de Trasplante de Medula Osea, Madrid, Spain; King Faisal Specialist Hospital and Research Center, Department of Oncology, Jeddah, Saudi Arabia; University of Amiens: CHU Amiens, Service d`Hematologie, Amiens, France; Hospital Clinic, Institute of Hematology & Oncology, Dept. of Hematology, Barcelona, Spain; S.S.C.V.D Trapianto di Cellule Staminali, A.O.U Citta della Salute e della Scienza di Torino, Torino, Italy; CHU CAEN, Institut d’hématologie de Basse-Normandie, Caen, France; Hospital Santa Creu i Sant Pau, Hematology Department, Barcelona, Spain; University Hospital Birmingham NHSTrust, Queen Elizabeth Medical Centre, Edgbaston, Dept. of Haematology, Birmingham, United Kingdom; Department of Bone Marrow Transplantation and Oncohematology, Maria Sklodowska-Curie National Research Institute of Oncology, Oncology Center, Gliwice, Poland; Anadolu Medical Center Hospital, Bone Marrow Transplantation Department, Kocaeli, Turkey; University Hospital, Department of Hematology and Transfusiology, Bratislava, Slovakia; ICO-Hospital Universitari Germans Trias i Pujol, Cattedra e Servizio di Ematologia, Badalona, Spain; University Hospital Eppendorf, Bone Marrow Transplantation Centre, Hamburg, Germany; CHU - Institut Universitaire du Cancer Toulouse, Oncopole, I.U.C.T-O, Toulouse, France; Charles University Hospital, Dept. of Hematology/Oncology, Pilsen, Czech Republic; Chaim Sheba Medical Center, Chaim Sheba Medical Center, Dept. of Bone Marrow Transplantation, Tel Hashomer, Israel; ¨Tor Vergata¨ University of Rome, Stem Cell Transplant Unit, Policlinico Universitario Tor Vergata, Rome, Italy; University Hospital, Clinic of Hematology, Zurich, Switzerland; Hopital Jean Minjoz, Service d`Hématologie, Besancon, France; Hospital U. Marqués de Valdecilla, Servicio de Hematología-Hemoterapia, Santander, Spain; Hope Directorate, St. James`s Hospital, Dublin, Ireland; Belfast City Hospital, Dept. of Haematology, Belfast, United Kingdom; Skanes University Hospital, Dept. of Hematology, Lund, Sweden; Ankara Bayindir Hospital, Haematology BMT, Eskisehir yolu Sogutozu, Ankara, Turkey; CHU Nice - Hôpital de l`ARCHET I, Hematologie Clinique, Nice, France; IRCCS, Casa Sollievo della Sofferenza, DEPARTMENT OF HEMATO-ONCOLOGY, STEM CELL TRANSPLANT UNIT, SGiovanni Rot, Italy; Universitaetsklinikum Goettingen, Abteilung Hämatologie und Onkologie, Goettingen, Germany; University Hospital Maastricht, Dept. Internal Med.Hematology /Oncology, Maastricht, The Netherlands; University Department of Hematology, Arhus Amtssygehus, Aarhus, Denmark; Hospital Clínico, Servicio de Hematología, Salamanca, Spain; Ospedale San Raffaele s.r.l., Haematology and BMT, Milano, Italy; Bone Marrow Transplant Unit L 4043, National University Hospital, Rigshospitalet, Copenhagen, Denmark; Medizinische Universitaet Wien, Klinik fuer Innere Medizin I, Knochenmarktransplantation, Vienna, Austria; Hopital La Miletrie, Head of the Bone Marrow TransplantUnit, Clinical Hematology, Poitiers, France; Inst. Português de Oncologia do Porto, BMT Unit, Porto, Portugal; Hannover Medical School, Department of Haematology, Hemostasis, Oncology, and Stem Cell Transplantation, Hannover, Germany; Azienda Ospedaliera Universitaria Careggi, Cell Therapy and Transfusion Medicine Unit, Firenze, Italy; Istituto Clinico Humanitas, Transplantation Unit, Department of Oncology and Haematology, Milano, Italy; Bristol Royal Hospital for Children, Dept. of Paediatric Oncology/BMT, Bristol, United Kingdom; Grande Ospedale Metropolitano Bianchi Melacrino Morelli - Centro Unico Trapianti A. Neri, Alberto Neri, Bianchi-Melacrino-Morelli, Reggio Calabria, Italy; University Med. Center, Department of Hematology, Ljubljana, Slovenia; University of Muenster, Dept. of Hematol./Oncol., Muenster, Germany; Institute of Hematology and Transfusion Medicine, I. Gandhi 14 str., Warsaw, Poland; National Research Center for Hematology, Bone Marrow Transplantation, Moscow, Russia; Centre Henri Becquerel, Hematology, Rouen, France; Universitaetsmedizin Mannheim, III. Medizinische Klinik, Einheit für Stammzelltransplantation, Mannheim, Germany; University College London Hospital, Department of Haematology, London, United Kingdom; Cliniques Universitaires St. Luc, Dept. of Haematology, Brussels, Belgium; Oslo University Hospital, Rikshospitalet, Clinic for Cancer Medicine, Hematology Dept., Section for Stem Cell Transplantation, Oslo, Norway; Ospedale S. Camillo-Forlanini, Dept. of Hematology and BMT, Rome, Italy; Rambam Medical Center, Dept. of Hematology & BMT, Haifa, Israel; University Medical Center Groningen (UMCG), Dept. of Hematology, Groningen, The Netherlands; ICO – Hospital Duran i Reynals, L`Hospitalet de Llobregat, Barcelona, Spain; H SS. Antonio e Biagio, Haematology Department, Alessandria, Italy; Universitaet Tuebingen, Medizinische Klinik, Tuebingen, Germany; Turku University Hospital, TD7 (Stem Cell Transplant Unit), Turku, Finland; Hôpital Henri Mondor, Sve d` Hematologie, Creteil, France; Département d`Oncologie, Service d`Hématologie, Hôpitaux Universitaires De Genève, Geneva, Switzerland; Medical University Graz, LKH - University Hospital Graz, Division of Haematology, Graz, Austria; Beilinson Hospital, Hematology and BMT Department, Petach Tikva, Israel; Fundeni Clinical Institute, 258 Fundeni Street, Building A, 7th Floor, Bucharest, Romania; VU University Medical Center, Department of Hematology (Br 250), Amsterdam, The Netherlands; University Hospital Brno, Dept. of Internal Med. - Hematooncology, Brno, Czech Republic; Ege University Medical School, Dept. of Hematology, Izmir, Turkey; Philipps Universitaet Marburg, University Hospital Giessen and Marburg, Marburg, Germany; U.O. Ematologia con Trapianto, Azienda Ospedaliero Universitaria Policlinico Bari, Bari, Italy; Hôpital Percy, Hematology Department, Clamart, France; Istanbul University-Cerrahpasa, Cerrahpasa Medical Faculty BMT Unit, Istanbul, Turkey; University of Napoli, `Federico II` Medical School, Division of Hematology, Napoli, Italy; Medizinische Klinik m. S. Hämatologie , Onkologie und Tumorimmunologie, Charité Universitätsmedizin Berlin, Berlin, Germany; Antwerp University Hospital (UZA), Dept. of Hematology, Antwerp Edegem, Belgium; Hôpital Necker, Service Hematologie Adulte, Paris, France; Centre National de Greffe de Moelle, Rue Jebel Lakhdar, Tunis, Tunisia; Institut Jules Bordet, Experimental Hematology, Brussels, Belgium; Univ. La Sapienza, Dip. Biotecnologie Cellulari ed Ematologia, Rome, Italy; University Hospital, Dept. of Medicine, Uppsala, Sweden; University Hospital Innsbruck, Internal Medicine V (Hematology & Oncology), Innsbruck, Austria; CHU ESTAING, Service d’hématologie clinique Adulte et pédiatrie, Clermont Ferr, France; Fundación Jiménez Díaz, Hematología, Madrid, Spain; European Institute of Oncology, Institute of Haematology, Milano, Italy; Klinikum Rechts der Isar, III Med Klinik der TU, Munich, Germany; Hospital Vall d`Hebron, Unidad de Adultos, Barcelona, Spain; Manchester Royal Infirmary, Clinica Haematology Department, Manchester, United Kingdom; Policlinico G.B. Rossi, Divisione di Ematologia, Unità di TMO, Verona, Italy; CHRU, Service des Maladies du Sang, Angers, France; Ospedale La Maddalena - Dpt. Oncologico, Unità Operativa di Oncoematologia e, Trapianto di Midollo, Palermo, Italy; University Medical Center Mainz, Department of Hematology, Oncology and Pneumology,, Mainz, Germany; A.O.R.N. `SAN.G MOSCATI`, Ematologia, Avellino, Italy; Medical University of Gdansk, University Hospital, Dept. of Haematology and Transplantology, Gdansk, Poland; Robert_Bosch_Krankenhaus, Abt. Hämatologie / Onkologie, Stuttgart, Germany; University Medical Centre, Dept. of Haematology, Utrecht, The Netherlands; Bologna University, S.Orsola-Malpighi Hospital, Institute of Hematology & Medical, Oncology L & A Seràgnoli, Bologna, Italy; Bone Marrow Transplant Unit, Beatson, West of Scotland Cancer Centre, Gartnaval General Hospital, Glasgow, United Kingdom; Institut de Cancerologie Lucien Neuwirth, Service d`Hematologie Clinique, Saint Etienne, France; Yorkshire Blood & Marrow Transplant Programme, Haematology Department, Level 3, Bexley Wing, St James`s Institute of Oncology, Leeds, United Kingdom; Fondazione IRCCS - Ca’ Granda, Ospedale Maggiore Policlinico IRCCS, Milano, Italy; Hopital Bretonneau, Service d`Oncologie Médicale, Tours, France; U.O.S.A Centro Trapianti e Terapia Cellulare, Azienda Ospedaliera Universitaria Senese, Policlinico S.Maria alle Scotte, Siena, Italy; AORMN Hospital, Hematology & Transplant Centre, Pesaro, Italy; Azienda Ospedaliero Universitaria di Modena Policlinico, Ematologia, Modena, Italy; NADACE HAIMOM, University Hospital, Department of Haemato-Oncology, Olomouc, Czech Republic; Elisabethinen-Hospital, I. Internal Department, Linz, Austria; Hospital Ramón y Cajal, Servicio de Hematología, Madrid, Spain; Ankara University Faculty of Medicine, Dept. of Hematology, Adult Stem Cell Transplantation Unit, Ankara, Turkey; C.H.R.U de Brest, 2, avenue Foch, Service Onco-Hematologie, Brest, France; Silesian Medical Academy, Univ. Dept. of Haematology and BMT, Katowice, Poland; University Regensburg, Dept. of Hematology and Oncology, Regensburg, Germany; University Hospital Erlangen, Dept. of Internal Medicine 5, Erlangen, Germany; Onco-Ematologia Pediatrica, Centro Trapianti Cellule Staminali, Ospedale Infantile Regina Margherita, Torino, Italy; Ospedale Dell`Angelo, Hematology Department, Venezia, Italy; A.Z. Sint-Jan, Dept. of Hematology, Brugge, Belgium; HELIOS Klinikum Berlin-Buch, Klinik für Hämatologie und Stammzelltransplantation, Berlin, Germany; University of Cologne, I. Dept. of Medicine, Cologne, Germany; Hospital Univ. Virgen de las Nieves, Servicio de Hematología, Granada, Spain; Addenbrookes Hospital, Department of Haematology, Cambridge, United Kingdom; Hospital Clinico Universitario, Servicio de Hematología, S de Compostela, Spain; Clinic of Hematology, Military Medical Academy, Belgrade, Serbia and Montenegro; Fiona Stanley Hospital, Hematology Department, Perth, Australia; University of Liege, Dept. of Hematology, CHU Sart-Tilman, Liege, Belgium; Clinica Puerta de Hierro, Servicio de Hematologia y Hemoterapia, Madrid, Spain; Ghent University Hospital, Haematology, Gent, Belgium; St. Bartholomew`s and The Royal London NHS Trust, West Smithfield, London, United Kingdom; Christie NHS Trust Hospital, Adult Leukaemia and Bone Marrow Transplant Unit, Manchester, United Kingdom; Azienda Ospedali Riuniti di Ancona, Department of Hematology, Ancona University, Ancona, Italy; Ospedale Policlinico, Programma di Trapianto Emopoietico Misto e Metropolitano Di Catania, Catania, Italy; IRRCS Ospedale Pediatrico Bambino Gesù, Piazza S. Onofrio, 4, Rome, Italy; Medstar Antalya Hospital, Stem Cell Transplantation Unit, Antalya, Turkey; Unita Operativa di Ematologia e Trapianto di cellule staminali, Presidio Ospedaliero Vito Fazzi, Lecce, Italy; FOSCAL-UNAB, Urbanización El Bosque, Floridablanca, Colombia; Asklepios Klinik St. Georg, Department of Haematology, Hamburg, Germany; Hospital Guglielmo da Saliceto, Oncology and Hematology Department, Piacenza, Italy; Klinikum Frankfurt (Oder) GmbH, Medizinische Klinik I, Frankfurt Oder, Germany; Royal Marsden Hospital, Leukaemia Myeloma Units, London, United Kingdom; University Medical Center Schleswig-Holstein, Campus Kiel, División of Stem Cell Transplantation and Immunotherapy, Kiel, Germany; Klinikum Karlsruhe gGmbH, III. Med. Klinik, Haematologie, Onkologie, Karlsruhe, Germany; Hopital d`Enfants de la Timone, CHU, Département Hématologie Oncologie Pédiatrique, Marseille, France; Department of Haematology, University Hospital of Wales, Cardiff, United Kingdom; University Hospital Aachen, Dept. of Oncology, Hematology and SCT, Medizinische Klinik IV, Aachen, Germany; Universitaetsklinium Magdeburg, Med. Fakultät d., Klin.f.Hämatol./Onkologie, Magdeburg, Germany; Complejo Hospitalario de A Coruña, Hematologia (Planta 11ª), La Coruna, Spain; Heinrich Heine Universitaet, Klinik für Hämat,Onkol,Klin.Immun., Duesseldorf, Germany; Singapore General Hospital, Singapore, Singapore; King Fahad Specialist Hospital, Adult Hematology and HSCT department, Dammam, Saudi Arabia; ALBERTS CELLULAR THERAPY, Netcare Pretoria East Hospital, Pretoria, South Africa; Clatterbridge Cancer Centre - Liverpool, Royal Liverpool University Hospital, Clatterbridge Cancer Centre NHS Foundation Trust, Division of Stem Cell Transplantation and Haemato-, Liverpool, United Kingdom; DCTK, ul. Grabiszynska 105, Marrow Donor Registry, Wroclaw, Poland; National Haematology Centre, Clinic Linezers, Riga, Latvia; Arcispedale S. Maria Nuova, Unita Operativa Ematologia, Reggio Emilia, Italy; Osmangazi University, Fac. of Medicine, Hasan Dolatkan Street, Eskisehir, Turkey; Ankara Sehir Hastanesi, Universiteler mahallesi Bilkent caddesi no 1, Ankara, Turkey; Samodzielny Publiczny, Szpital Kliniczny Nr 1 w Lublinie, Klinika Hematoonkologii i Transplantacji Szpiku,, Lublin, Poland; Southampton General Hospital, Haematology, Oncology, & Paediatrics, Dept. of Haematology, Southampton, United Kingdom; Leicester Royal Infirmary, Department of Haematology, NHS Trust, Leicester, United Kingdom; Nottingham University, Hucknall Road, Nottingham, United Kingdom; University Hospital, Dept. of Hematology, Linkoeping, Sweden; Klinikum Oldenburg, Abt. Onkologie/Hämatologie, Oldenburg, Germany; Ýstanbul Tip Fakultesi, Iç Hastaliklari ABD, Kemik iliði nakil unitesi, CAPA, Istanbul, Turkey; Sezione di Ematologia, Dipartimento di Medicina Clinica e Sperimentale, Università di Perugia, Ospedale Santa Maria della, Perugia, Italy; S. Bortolo Hospital, Department of Hematology, Vicenza, Italy; Universitaetsklinikum Dresden, Medizinische Klinik und Poliklinik I, Dresden, Germany; University of Freiburg, Dept. of Medicine -Hematology, Oncology, Freiburg, Germany; Private Medicana International Ankara Hospital, Dept. Bone Marrow Transplantation, Ankara, Turkey; Central Clinical Hospital, The Medical University of Warsaw, Department of Hematology & Oncol*, Warsaw, Poland; St. Franziskus Hospital, Medizinische Klinik I, Flensburg, Germany; Mazzoni Hospital, Haematology Service, Ascoli Piceno, Italy; Haukeland University Hospital, Department of Haematology, Bergen, Norway; Klinik fuer Innere Medzin III, Universitätsklinikum Ulm, Ulm, Germany; Nijmegen Medical Centre, Department of Hematology, Nijmegen, The Netherlands; Adult HSCT unit, Northern Centre for Bone Marrow Transplantation, Freeman Hospital, Newcastle Tyne, United Kingdom; Centro Trapianti di Midollo Osseo, Clinica Pediatrica Università di Milano Bicocca, Fondazione MBBM - Ospedale San Gerardo, Monza, Italy; Inst. Portugues Oncologia, BMT Unit, Lisboa, Portugal; Martin-Luther-Universitaet Halle-Wittenberg, Klinik für Innere Medizin IV, Halle, Germany; Medical Clinic and Policinic 1, Hematology and Cellular Therapy, University hospital Leipzig, Johannisallee 32 A, Leipzig, Germany; Peking University People´s Hospital, Institute of Haematology, Beijing, China; Istanbul Medipol University,, Medipol Mega Hospital Complex, Istanbul, Turkey; Hospital de Gran Canaria `Dr Negrin`, Servicio de Hematología y Hemoterapia, Las Palmas, Spain; St. George`s Hospital, Department of Haematology, London, United Kingdom; Jagiellonian University, Department of Haematology, Krakow, Poland; Hospital Regional de Málaga, Servicio de Hematología, Malaga, Spain; Belarussian Research Center for Pediatric Oncology, Hematology and Immunology, Frunzenskaya str., 43, Minsk, Belarus; Hospital Universitario Donostia, Paseo Dr Beguiristain 107-116, San Sebastian, Spain; Klinikum Bremen-Mitte, Hämatologie / Onkologie, Klinik für Innere Medizin, Bremen, Germany; Az. Ospedaliera S. Croce e Carle, Division of Hematology, Cuneo, Italy; Hospital del SAS, Dept. of Hematology, Cadiz, Spain; Klinikum Nuernberg, 5. Medizinische Klinik, BMT-Unit, Nuernberg, Germany; Vilnius University Hospital Santaros Klinikos, Haematology, Oncology & Transfusion Center, Vilnius, Lithuania; AZ Delta, Hematology - Oncology Dept., Roeselare, Belgium; Pediatric University Teaching Hospital, BMT Unit, II Children`s Clinic, Bratislava, Slovakia; Hospital Universitari Son Espases, Hematology Service, Palma Mallorca, Spain; Charles University Hospital, 4th Department of Internal Medicine - Hematology, Hradec Kralove, Czech Republic; Tartu University Hospital, Clinic of Hematology and Oncology, Tartu, Estonia; Kings College Hospital, Dept. of Haematological Medicine, King`s Denmark Hill Campus, London, United Kingdom; University Hospital, Collegium Medicum UMK, Pediatric Hematology and Oncology, Bydgoszcz, Poland; Sheffield Teaching Hospitals NHS Trust, South Yorkshire Region (Adult) BMT Programme, Royal Hallamshire Hospital , Sheffield, United Kingdom; University of Saarland, University Hospital, Dept. of Internal Med., BMT Unit, Homburg, Germany; Azienda Ospedaliero Universitaria Pisana, Unità Operativa Ematologia, Pisa, Italy; Federal Centre of Heart, Blood and Endocrinology, named after V.A. Almazov, Dept. of Hematology, St Petersburg, Russia; CHRU Limoges, Service d`Hématologie Clinique, Limoges, France; Ospedale dei Bambini, Paediatric Haematology - Oncology, Palermo, Italy; ZSIS Universitaetsklinikum Knappschaftskrankenhaus Bochum GmbH, Medizinische Klinik - Hämatologie und Onkologie, Bochum, Germany; Universitaet Bonn, Medizinische Klinik III, Bonn, Germany; Diakonissenkrankenhaus, Med. Klinik Abteilung 2, Stuttgart, Germany; Klinikum Augsburg, II Medizinische Klinik, Augsburg, Germany; Tel Aviv Sourasky Medical Center, Blood and Bone Marrow Transplantation, Tel Aviv, Israel; Karadeniz Technical University, Faculty of Medicine, Department of Haematology, Trabzon, Turkey; National Children`s Specialized Hospital OCHMATDYT, Center of Pediatric Oncohematology and BMT, BMT-department, Kiev, Ukraine; Adnan Menderes University Med. Faculty, Hematology Department, Aydin, Turkey; Department of Haematology, Cancer and Haematology Centre, Churchill Hospital, Oxford, United Kingdom; Hospital Clínico de Valencia, Servicio de Hematología, Valencia, Spain; Birmingham Heartlands Hospital, Department of Haematology, Birmingham, United Kingdom; Clinica di Oncoematologia Pediatrica, Dipartimento di Pediatria, Padova, Italy; Sahlgrenska University Hospital, Center for Hematopoietic Cell Transplantation, Hematology Section, Goeteborg, Sweden; Hospital San Maurizio, Dept. of Hematology - BMT Unit, Bolzano, Italy; Deutsche Klinik fuer Diagnostik, KMT Zentrum, Wiesbaden, Germany; Hospital Sao Joao, Servicio de Hematologia Clinica, Porto, Portugal; GATA BMT Center, Gülhane Military Medical Academy, Ankara, Turkey; Hospital Univ. 12 de Octubre, Servicio de Hematología, Madrid, Spain; Gaziantep University Medical School, Division of Haematology, Gaziantep, Turkey; Makassed University Hospital, Makassed University Hospital, Haematology/Oncology, Beirut, Lebanon;

Hospital Álvaro Cunqueiro - Complejo Hospitalario Universitario de Vigo, Servicio de Hematología, Vigo, Spain; Istanbul Medipol University,, Medipol Mega Hospital Complex, Istanbul, Turkey; National Center for Cancer Care & Research, Hematology, Doha, Qatar; Universitaetsklinikum Muenster, Klinik für Kinder- und Jugendmedizin, Pädiatrische Hämatologie und Onkologie , Muenster, Germany; University of Cape Town Faculty of Health Sciences, Division of Clinical Haematology, Cape Town, South Africa; Unidad de Ensayos Clínicos de Hematología Pabellón A, bajo., Complejo Hospitalario de Navarra, Pamplona, Spain; Universitaet Rostock, Kl. für Inn.Med./Hämatologie/Onkol., Rostock, Germany; University of Milano, Istituto Nazionale dei Tumori, Hematology - Bone Marrow Transplantation Unit, Milano, Italy; Erciyes Medical School, Dept. of Hematology - Oncology, Kapadokya (Cappadocia) BMT Center, Kayseri, Turkey; Hospital Universitario Central de Asturias, Avenida de Roma S/N, Oviedo, Spain; Dokuz Eylul University, School of Medicine, Department of Hematology, Izmir, Turkey; Uniwersytecki Szpital Kliniczny, Department of Hematology, Wroclaw, Poland; Universitaetsklinikum Wuerzburg, Med. Klinik und Poliklinik II, Wuerzburg, Germany; Umea University Hospital, Hematology, Umea, Sweden; Hospital Universitario La Paz, Hematologia-Oncologia, Madrid, Spain; Hospital Morales Meseguer, C/ Marqués de los Velez s/n, Unidad de Trasplante de Médula Osea, Serv de Hemat, Murcia, Spain; Clínica Universitaria de Navarra, Area de Terápia Celular, Unidad de Trasplante Hemopoyético, Pamplona, Spain; Haematology Department, St.Savvas Oncology Hospital, Athens, Greece; Evangelisches Krankenhaus Essen-Werden gGmbH, Haematologie/Onkologie/Stammzelltransplantation, Essen, Germany; Canterbury Health Laboratories, Department of Haematology, Christchurch, New Zealand; Institut d`Hematologie et d`Oncologie Pediatrique, 1 Place Professeur Joseph Renaut, Lyon, France; Cape of Hope, Wroclaw Medical University, Department of Pediatric Bone Marrow Transplantatio, Wroclaw, Poland; University Hospitals Plymouth NHS Trust, Derriford Hospital, Plymouth, United Kingdom; National Hospital of Haematological Diseases, Bone Marrow Transplant, Sofia, Bulgaria; Ospedale San Carlo, Dip. Ematologia, Potenza, Italy; University Hospital of Oran, Department of Haematology and Cell Therapy, USTO, Oran, Algeria
