# Supplementary material for: Outcome of haploidentical versus matched sibling donors in hematopoietic stem cell transplantation for adult patients with acute lymphoblastic leukemia: a study from the Acute Leukemia Working Party of the European Society for Blood and Marrow Transplantation
Source: J Hematol Oncol. 2021 Apr 1;14:53. doi: 10.1186/s13045-021-01065-7 (PMC8017786; doi:10.1186/s13045-021-01065-7)
Supplement: Supplementary file 2 — Additional file 2: Supplementary tables. [file 13045_2021_1065_MOESM2_ESM.docx]

**Supplementary table 1.** Conditioning

**Abbreviations:** Bu-Busulfan; Cy-Cytoxan; Vp16/Eto-Etoposid;Fluda/Flu-Fludarabine; ARAC/ARAC-cytosar ;TBF-Thiotepa/Busulfan/Fludarabine;Mel-Melphalan;BCNU-Carmustine;Treo-Treosulfan;TBI-Total body irradiation;Flamsa-fludarabine, amsacrine, and cytarabine ;Clo-Clofarabine; Sequential Thio-ETO-BuFlu-Cy/ Clofa-ARAC-Bu2-Cy-

**Supplementary Table 2.** Causes of death

**Abbreviations:** VOD- veno-occlusive disease of the liver; IP- interstitial pneumonitis; GVHD-graft versus host disease; MOF- multi organ failure; transp-transplantation

**Supplementary table 3.** Matched pair analysis -patient, donor, disease and transplant characteristics

Abbreviations: KPS, Karnofsky performance status;TBI, total body irridiation; CMV, cytomegalovirus

**Supplementary table 4.** Matched pair analysis - Transplant characteristics

Abbreviations: MSD: matched sibling donor, Haplo:haploidentical transplantation , MAC: myeloabalative conditioning, RIC: reduced intensity conditioning, TBI:total body irradiation ;BM-bone marrow; PB-mobilized peripheral blood stem cells; PTCy-post transplantation cyclophosphamide; ATG-anti thymocyte globulin; MAC- myeloablative conditioning; TBI-total body irradiation; CT-chemotherapy; RIC-reduced intensity conditioning

**Supplementary table 5.** Matched pair analysis - Transplant outcome

Abbreviations: HSCT-Hematopoietic stem cell transplantation; GVHD-graft versus host disease

**Supplementary table 6.** Matched pair analysis of clinical outcomes

Abbreviations: RI, relapse incidence; NRM, non-relapse mortality; LFS, leukemia-free survival; OS, overall survival; GVHD, graft versus host disease; GRFS, GVHD-free/relapse-free survival; KPS, Karnofsky performance status; ATG, anti-thymocyte globulin; PTCy, post-transplant cyclophosphamide; RIC, reduced intensity conditioining; MAC, myeloablative conditioning; PB, peripheral blood; BM, bone marrow

**Supplementary table 7.** Matched pair analysis - Causes of death

**Abbreviations:** VOD- veno-occlusive disease of the liver; IP- interstitial pneumonitis; GVHD-graft versus host disease; MOF- multi organ failure; transp-transplantation
